# Hemodynamic phenotyping of bronchopulmonary dysplasia: from transitional circulation to precision cardiopulmonary care

**DOI:** 10.3389/fped.2026.1877119

**Published:** 2026-06-25

**Authors:** Gabriela S. Trindade, Bianca C. Benincasa, Rita C. Silveira, Renato S. Procianoy

**Affiliations:** Department of Pediatrics, Newborn Section, Hospital de Clínicas de Porto Alegre, Universidade Federal do Rio Grande do Sul, Porto Alegre, RS, Brazil

**Keywords:** bronchopulmonary dysplasia, hemodynamics, natriuretic peptide, brain, patent ductus arteriosus, premature infant, preterm birth, targeted neonatal echocardiography

## Abstract

**Objectives:**

Bronchopulmonary dysplasia (BPD) remains one of the most important complications of extreme prematurity and a leading cause of long-term respiratory, cardiovascular, and neurodevelopmental morbidity. Increasing evidence suggests that BPD should be viewed not only as a parenchymal lung disorder but as a complex cardiopulmonary syndrome involving disrupted vascular development, abnormal transitional circulation, and ventricular dysfunction. This review aimed to summarize current evidence on the hemodynamic mechanisms underlying BPD, emphasizing pulmonary vascular disease (PVD), bronchopulmonary dysplasia-associated pulmonary hypertension (BPD-PH), phenotype-based classification, and implications for precision management.

**Methods:**

A narrative review of experimental, translational, and clinical studies was performed, focusing on pulmonary vascular development, transitional hemodynamics, patent ductus arteriosus, ventricular function, targeted neonatal echocardiography, and biomarker-based risk stratification in preterm infants. Evidence regarding phenotypic classification and individualized therapeutic strategies was also examined.

**Results:**

Emerging evidence demonstrates that abnormal pulmonary vascular growth begins early, often during the transitional circulatory period, and is aggravated by hyperoxia, mechanical ventilation, inflammation, placental dysfunction, and altered pulmonary blood flow. Prolonged exposure to hemodynamically significant left-to-right shunts, particularly patent ductus arteriosus, may contribute to pulmonary overcirculation, edema, and vascular remodeling. Elevated pulmonary vascular resistance leads to right ventricular pressure overload, while left ventricular diastolic dysfunction and pulmonary venous congestion further worsen pulmonary edema and gas exchange. Early hemodynamic assessment using targeted neonatal echocardiography and biomarkers such as NT-proBNP enables detection of subclinical PVD and ventricular dysfunction during the first days of life. Phenotype-based classification reveals overlapping parenchymal, interstitial, congestive, vascular, and airway components, supporting individualized cardiopulmonary management.

**Conclusions:**

BPD is increasingly recognized as a heterogeneous cardiopulmonary syndrome in which disturbed hemodynamics and impaired cardiopulmonary coupling play central roles in disease progression and prognosis. Early hemodynamic phenotyping may improve risk stratification, support precision-guided interventions, and offer new opportunities to prevent PVD, BPD-PH, and long-term cardiopulmonary sequelae in extremely preterm infants.

## Introduction

BPD remains the major complication of extreme prematurity and a key cause of long-term cardiopulmonary and neurodevelopmental morbidity, leading to recurrent respiratory hospitalizations, pulmonary hypertension (PH) and impaired growth and neurodevelopment. BPD-PH develops in approximately 25% of infants with moderate-to-severe BPD and is strongly associated with poor outcomes, including early mortality and higher respiratory morbidity (1–3).

Since its original description, the condition has undergone a marked epidemiological and pathological transition. The earlier form of BPD, typically observed in more mature preterm infants exposed to high concentrations of supplemental oxygen and invasive mechanical ventilation, was characterized by airway injury, inflammation, and fibroproliferative remodeling. In contrast, the contemporary form predominantly affects infants born at earlier gestational ages and is defined by disrupted lung development, including simplified alveolar architecture and abnormal pulmonary vascular growth. Consequently, BPD is understood as a vasculo-alveolar disease, in which impaired angiogenesis, abnormal pulmonary microvascular development and hemodynamic stress drive pulmonary vascular disease (PVD), a progressive rise in pulmonary vascular resistance (PVR) and, eventually, PH (1, 3, 4). In parallel, a hemodynamic perspective has gained prominence, highlighting how abnormal pulmonary blood flow, chronic pressure and volume overload of the right (RV) and left (LV) ventricles and disturbed cardiopulmonary coupling are central to disease progression, particularly in BPD-PH (5–7).

In this review, BPD will be examined from this integrated hemodynamic perspective, focusing on altered transitional circulation and the onset of PVD, the impact of pulmonary overcirculation, particularly due to patent ductus arteriosus (PDA), vascular remodeling and PH, cardiopulmonary coupling, the role of functional echocardiography and targeted hemodynamic assessment in early risk stratification, and phenotyping of BPD-PH, and individualized management. By linking pathophysiological mechanisms with hemodynamically guided interventions, the aim is to contribute to a more precise approach to the prevention, treatment, and prognosis of BPD in extremely preterm neonates, in line with current research priorities in the field.

## Literature search

A literature search was performed across major databases, including PubMed and Medline. Keywords such as “Premature infant”, “Preterm Birth”, “Bronchopulmonary Dysplasia,” “Hemodynamics”, “Patent Ductus Arteriosus,” “Targeted Neonatal Echocardiography”, “Natriuretic Peptide, Brain” and were used in various combinations, with the latest search conducted in April 2026. We used Ryyan to screen the studies. We included reviews, metanalyses, clinical trials, observational studies and experimental studies. We identified and screened 3647 studies. Only studies published in English were included. Additionally, references from relevant articles were screened to identify other pertinent studies not captured in the initial search. Recent articles were selected based on the authors' judgment regarding their relevance to the topic.

## Transitional circulation, angiogenesis, and the onset of PVD in BPD

The transition from fetal to neonatal circulation normally involves a rapid fall in PVR, increased pulmonary blood flow, and functional closure of fetal shunts. In extremely preterm infants, however, this transition occurs in a structurally and functionally immature lung still in a critical phase of organogenesis. Abrupt exposure to a high-oxygen environment, the need for mechanical ventilation, and frequent hemodynamic instability and infection impose substantial injury on a developing vascular bed (1, 3, 8).

BPD's core pathophysiology centers on abnormal pulmonary blood flow impacting an immature circulation. The pulmonary vasculature exhibits dysmorphic growth—characterized by a reduced number of small arteries and irregular interstitial organization—which diminishes the alveolar-capillary surface area and impairs gas exchange. Early lung injury from hyperoxia, mechanical ventilation, or inflammation further disrupts angiogenesis and alveolarization, perpetuating a cycle in which vascular abnormalities both contribute to and result from BPD progression (1, 8).

Even before birth, maternal and placental factors such as preeclampsia, placental dysfunction, and intrauterine growth restriction can impair development of the pulmonary vascular bed, reducing fetal angiogenic capacity and predisposing to PVD from intrauterine life onwards. After birth, additional insults intensify mechanical and oxidative stress on the pulmonary circulation, maintain PVR at high levels, interrupt vascular growth, and favor the early establishment of PVD and PH (3, 5–7).

At this transitional stage, the components that will characterize established BPD are already emerging: arrested vascular growth with microvascular rarefaction and disorganization; failure of angiogenesis and alveolarization in a self-perpetuating vicious circle; and sustained elevation of PVR, which makes the pulmonary circulation highly vulnerable to even small increases in flow or episodes of hypoxia. Essentially, the origins of BPD and BPD-PH lie very early, at the interface between a disordered transitional circulation and a developing lung exposed to hemodynamic conditions for which it is not adequately prepared.

## Overcirculation, PDA, and left-to-right shunts

Among the early hemodynamic factors of BPD, pulmonary overcirculation due to left-to-right shunts plays a role. The presence of a hemodynamically significant PDA (hsPDA), as well as septal defects such as atrial or ventricular communications, leads to a substantial increase in blood flow to the immature lung (3, 5, 7, 9).

In PDA, blood is diverted from the aorta to the pulmonary artery, causing pulmonary hypercirculation, congestion, and edema, while reducing systemic blood flow to organs such as the kidneys, intestines, and brain (3, 10, 11). This overcirculation overwhelms the immature lung's lymphatic capacity and vascular compliance, leading to fluid leakage into the interstitium and subsequent interstitial and alveolar edema. The resulting thickening of the alveolar-capillary barrier impairs gas exchange, prompting increased oxygen and ventilatory support, which further exposes the lung to hyperoxia and baro/volutrauma.

The risk of BPD and BPD-PH increases with both the magnitude and duration of ductal shunting. Infants with moderate-to-large PDA persisting beyond 7–14 days are particularly likely to develop BPD, especially when invasive ventilation is required for more than 10 days (12, 13). Moreover, each additional month of PDA exposure, even with conservative management, is associated with a marked rise in BPD-PH risk, with adjusted odds ratios of approximately 4 for both any PDA and moderate-to-large PDA (10).

However, randomized clinical trials in human preterm infants have not consistently demonstrated that routine early PDA closure (<7 days of life) with non-steroidal anti-inflammatory drugs (indomethacin, ibuprofen, paracetamol) reduces BPD rates, even though such interventions are effective in closing the duct (11, 13, 14). A recent meta-analysis showed that pharmacological PDA treatment in the first two weeks of life, compared with expectant management, was associated with an increase in the composite outcome of death or moderate-to-severe BPD (RR: 1.10; 95% CI: 1.01–1.19) and with a nonsignificant increase in moderate-to-severe BPD alone (RR: 1.08; 95% CI: 0.95–1.23) (15).

This discrepancy between experimental and clinical data may reflect both the unintended effects of the intervention and the underlying hemodynamic context. Cyclo-oxygenase inhibitors can markedly reduce urine output and systemic blood flow, worsening fluid retention and pulmonary edema in the short term, while surgical ligation provokes an intense systemic inflammatory response with potential negative consequences for alveolar growth and lung fluid clearance (9). Moreover, the benefit of PDA closure appears to depend on individual physiology, as in some infants the ductus may transiently act as a “pop-off valve” for high pulmonary pressures, so indiscriminate closure can be harmful.

Thus, although there is a strong physiological rationale for targeting PDA as relevant therapeutic goal, clinical evidence suggests that management must be individualized, considering the specific hemodynamic profile and the risk of PH and ventricular failure. Prolonged patency of a hemodynamically significant ductal shunt nonetheless remains an important contributor to PVD in very and extremely preterm infants, underscoring the need for careful monitoring of PH signs and for explicit consideration of hemodynamic risk in PDA treatment decisions (3, 15). However, causality must not be overstated, routine early PDA closure have not consistently improved BPD outcomes, underscoring that association does not equal causation and that treatment decisions must remain individualized and hemodynamically guided.

## Pulmonary vascular remodeling and BPD-associated pulmonary hypertension

BPD-PH is characterized by three main vascular abnormalities: interruption of vascular growth; abnormal structural remodeling of the vascular wall; and increased vascular tone and reactivity (1–3, 8).

At the cellular and molecular level, hypertensive vascular remodeling in BPD-PH is driven by several key mechanisms. Hyperoxia triggers endothelial-to-mesenchymal transition (EndoMT) in pulmonary microvascular endothelial cells, promoting vascular wall thickening and pulmonary hypertension (16). Smooth muscle cell proliferation and fibroblast incorporation further increase medial hypertrophy and vascular stiffness (1). Additionally, impaired endothelial nitric oxide synthase (eNOS) phosphorylation reduces nitric oxide production and vasodilation, while enhanced calcium signaling in smooth muscle cells heightens vascular tone and vasoconstrictor responses (17).

Functionally, the result is chronically elevated PVR and abnormal vasoreactivity. Patients with BPD-PH exhibit marked vasoconstrictor responses even to mild hypoxia, as demonstrated in cardiac catheterization studies showing steep rises in pulmonary arterial pressure in response to small decreases in oxygen saturation, including in children with only moderate baseline PH (1, 17). Furthermore, increased vascular tone and endothelial dysfunction may persist into later childhood, suggesting that part of the vascular dysfunction is structural and not fully reversible (1).

Arrested vascular growth limits the ability of the pulmonary vascular bed to accommodate increased flow, leading to further rises in PVR. In this setting of critically reduced vascular reserve, even small left-to-right shunts can produce substantially greater hemodynamic overload than in infants with normal vascular development.

The PVD process that underlies BPD-PH results from multiple maternal, placental, fetal, and postnatal insults. Evidence suggests that this process develops progressively from birth and possibly begins already in fetal life (3, 5). Early injury in the developing lung sets up a vicious cycle in which impaired angiogenesis reduces alveolarization, while abnormal alveolar development further constrains vascular growth.

## Cardiopulmonary coupling: right and left ventricular dysfunction

In BPD-PH, chronically elevated PVR imposes sustained pressure overload on the RV, so that an initially adaptive hypertrophic response gradually evolves into maladaptive remodeling with progressive dilation, loss of contractility and overt RV failure. This pressure overload leads to reduced systolic performance and cardiac output, increased venous and pulmonary capillary pressures with congestion and edema, and may increase the risk of sudden death during acute decompensation (1, 2).

Ventricular interdependence amplifies the impact of this failure: RV dilation shifts the interventricular septum toward the LV, compromising LV filling and diastolic compliance and further reducing systemic output (5, 7). Early echocardiographic markers of RV impairment, such as reduced Tricuspid annular plane systolic excursion (TAPSE), lower RV fractional area change (FAC), and worsening RV myocardial performance index (MPI), have been described as strong predictors of BPD, BPD-PH, and mortality in extremely preterm infants (18). Thus, the RV is an integral component of self-perpetuating cycle of elevated PVR, vascular remodeling and pump failure.

## Left ventricle, venous congestion, and pulmonary edema

Emerging evidence shows that dysfunction, particularly diastolic dysfunction, and pulmonary venous congestion play an important role in the pathophysiology of BPD and its associated PH (1, 19, 20). The immature LV myocardium is particularly vulnerable to the hemodynamic stresses of the postnatal period, marked by abrupt changes in preload, systemic afterload, and pulmonary blood flow patterns. This scenario favors the development of both systolic and diastolic dysfunction and alters ventricular-arterial coupling (21). In infants with severe BPD, a high prevalence of elevated pulmonary capillary wedge pressure (PCWP) has been observed, suggesting that LV dysfunction or left atrial hypertension leads to pulmonary venous congestion and worsens edema (5, 7).

In addition, pulmonary vein stenosis, an acquired post-capillary obstruction, is increasingly recognized in preterm infants with BPD and PH, and is associated with recurrent pulmonary edema, refractory PH, and high mortality, although its true prevalence remains uncertain (1, 5, 7).

Functionally, around 25% of infants with BPD-PH show hemodynamic evidence of LV diastolic dysfunction on cardiac catheterization (1). Clinically, this often manifests as a persistent need for diuretic therapy to manage pulmonary edema, even when PH is only mild to moderate.

Serial echocardiographic studies have reinforced the importance of LV diastolic dysfunction in this population. These studies consistently demonstrate a prolonged isovolumic relaxation time (IVRT) from 33 to 36 weeks postmenstrual age, indicating impaired LV relaxation, as well as an increased LV MPI, with values >0.40 showing high sensitivity for the diagnosis of PH. In addition, an elevated E/E' ratio on tissue Doppler, reflecting increased LV filling pressures, is associated with longer duration of respiratory support, prolonged oxygen requirement, and higher incidence of BPD. Diastolic strain imaging further reveals a pattern of reduced early diastolic strain with relatively increased late diastolic strain, suggesting greater dependence on atrial contraction for effective LV filling (19, 20, 22). The E/E' ratio at 28 days of life is independently associated with duration of respiratory support and risk of BPD, indicating that LV diastolic dysfunction develops early and contributes to ongoing lung disease (20).

In parallel, there is growing recognition that systemic hemodynamics can aggravate LV dysfunction in infants with severe BPD (22, 23). Infants with BPD and systemic arterial hypertension exhibit prolonged IVRT, reduced E/A ratio, elevated E/e', increased left atrial and LV end-diastolic volumes, and higher PVR index. In many of these cases, a portion of the increased pulmonary pressures may be secondary to pulmonary venous hypertension from LV dysfunction, rather than purely pre-capillary pulmonary arterial disease (22).

This complex interaction between the LV, the systemic circulation, and the pulmonary vascular bed reinforces the concept of BPD-PH as a disorder of altered cardiopulmonary coupling, in which the left heart and systemic vasculature are as relevant as the RV and pulmonary circulation.

## Early hemodynamic screening: functional echocardiography and biomarkers

### NT-proBNP as a systemic marker of myocardial stress

Hemodynamic stress on the myocardium caused by increased afterload, volume overload, and ventricular dysfunction leads to myocardial wall stretch and consequent release of natriuretic peptides, particularly NT-proBNP.

In the context of extreme prematurity and BPD, NT-proBNP stands out as a plasma biomarker capable of reflecting, in an integrated manner, the degree of hemodynamic overload on the heart. Studies have shown that persistently elevated NT-proBNP levels as early as the first and second weeks of life independently and early predict which preterm infants will progress to severe BPD, BPD-PH, or death (24–27).

A recent study aimed to develop and internally validate a predictive model for hsPDA in preterm infants using a machine learning approach. BNP was the strongest predictor, followed by gestational age, birth weight, and surfactant use. The study found that a BNP cutoff of 62 pg/mL yielded a sensitivity of 80% and specificity of 81% for detecting hsPDA (26).

The main advantage of NT-proBNP is its ability to capture the global systemic response to hemodynamic stress, whether resulting from increased PVR, a large PDA, LV systolic–diastolic dysfunction, or combinations of these factors. Persistently high levels can therefore function as a laboratory alarm signal that complements clinical and echocardiographic assessment, helping to identify, at an early stage, infants at high risk of progressive cardiopulmonary vascular disease.

## Targeted neonatal echocardiography (TnECHO)

TnECHO, performed within the first week of life and, in some protocols, between 12 and 18 h after birth, represents a paradigm shift in the approach to PVD and BPD. Whereas traditional guidelines focused on screening for PH and PVD around 36 weeks' postmenstrual age, early TnECHO allows detection of hemodynamic abnormalities and biomechanical failure before hemodynamic stress results in irreversible vascular remodeling (18, 28–31).

Studies have shown that the presence of persistent PH between 72 and 96 h of life is a strong predictor of death or development of BPD at 36 weeks (32). Similarly, echocardiographic markers of PH detected on day 7 of life significantly increase the risk of late BPD-PH (31). In a cohort of infants born at <32 weeks' gestation, a pulmonary artery systolic pressure (sPAP) ≥ 25 mmHg and an sPAP-to-systemic systolic blood pressure ratio ≥0.35 in the first week were proposed as cut-off values to define early PH, capable of predicting BPD and mortality earlier than conventional PH thresholds (29).

The predictive power of TnECHO relies on the early measurement of quantitative indices specific to RV mechanics and the pulmonary vasculature (18, 28, 30). The pulmonary artery acceleration time (PAAT) and its ratio to RV ejection time (RVET) reflect the afterload imposed on the RV. Shortening of these intervals as early as day 7 functions as a strong predictor of the subsequent development of BPD, BPD-PH, and mortality. PAAT was considered the most indicative parameter of early pulmonary hemodynamic alterations. In the first 7 to 10 days of life, preterm infants who subsequently developed BPD or died exhibited significantly lower PAAT values compared to infants without BPD, even after adjusting for gestational age and birth weight. As PAAT is inversely related to PVR, this finding points to early vascular remodeling (30).

TAPSE is a simple and robust marker of longitudinal RV systolic function; low TAPSE values in the first week are associated with in-hospital mortality and with the composite outcome of severe BPD-PH. Tissue Doppler and diastolic function indices, such as E'/A' velocities and the MPI, capture early systolic–diastolic dysfunction of both RV and LV, which is frequently present in very low birth weight infants. Abnormalities in these indices on day 7 have also been shown to predict progression to BPD-PH.

Beyond its prognostic value, the major advantage of early TnECHO is that it enables physiology-driven precision care rather than reliance solely on nonspecific clinical signs. Systematic hemodynamic screening protocols between 12 and 18 h of life have demonstrated the ability to identify distinct physiological profiles during the transitional period, such as acute PH, primary RV dysfunction, LV dysfunction, and PDA, in which standard treatment might be inadequate or even harmful.

By understanding the underlying physiology, the clinical team can avoid the routine use of purely vasoconstrictive agents in high-afterload states, instead preferring inotropes that improve cardiac performance without further increasing PVR; restrict the use of inhaled nitric oxide to preterm infants with echocardiographically confirmed PH; and decide more precisely when to treat on the PDA.

Implementation of early hemodynamic echocardiographic screening in some centers has been associated not only with improved risk stratification but also with reductions in composite outcomes of death and severe intraventricular hemorrhage, with a trend toward lower rates of severe BPD and necrotizing enterocolitis, likely through optimization of systemic and pulmonary perfusion within the first hours of life (21, 33).

## Phenotypic classification and directed therapeutic approaches

**Figure 1:**
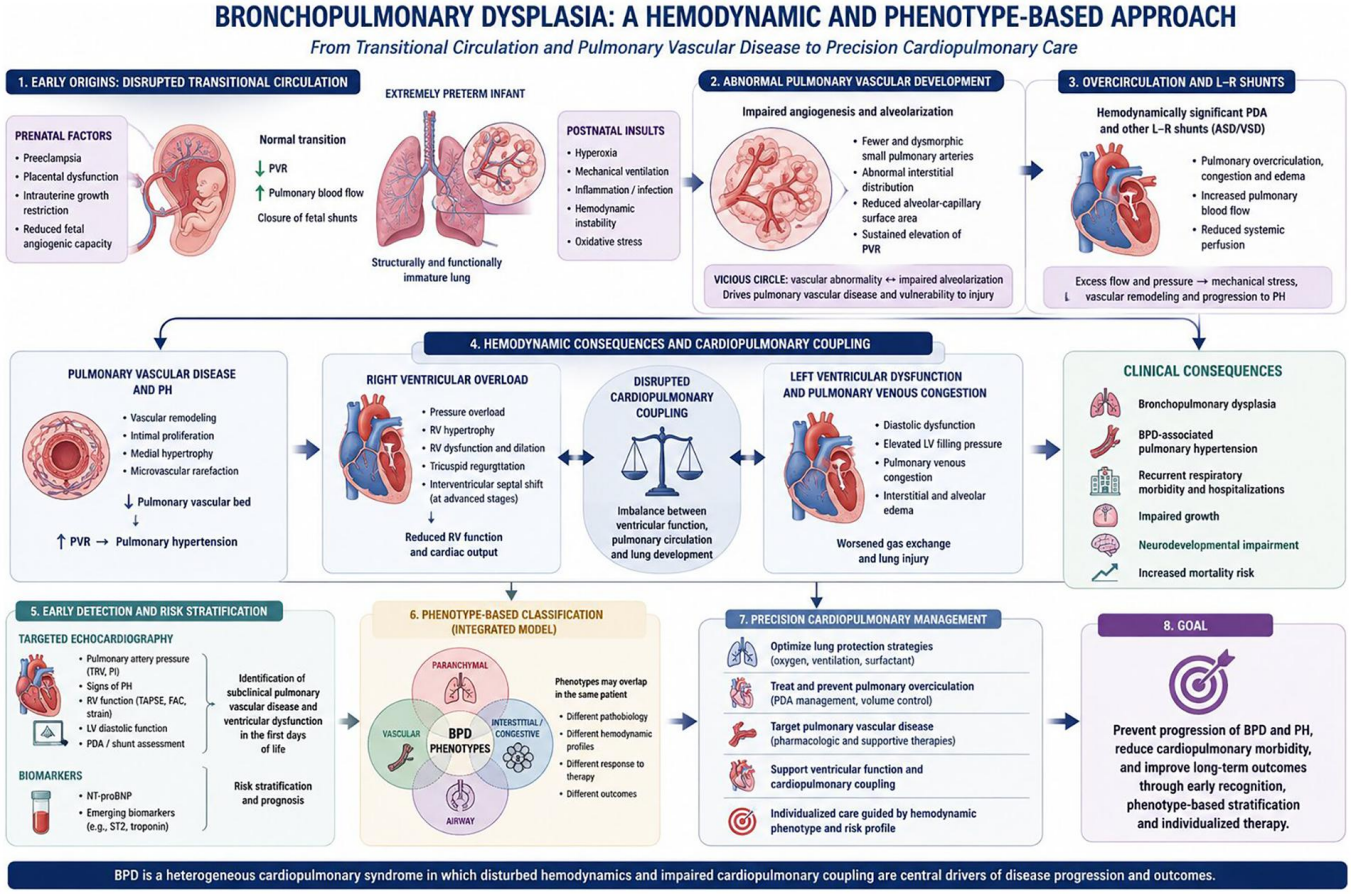
Bronchopulmonary dysplasia: hemodynamic and phenotype-bases approach. Image generated by AI and revised by the authors.

Despite advances in understanding the biological underpinnings of BPD, its clinical definition remains largely pragmatic and treatment-based, most anchored to the level of respiratory support required at 36 weeks PMA (34). While this approach has enabled consistency across clinical trials and epidemiological studies, it fails to adequately capture the substantial heterogeneity in underlying mechanisms. As a result, conditions with distinct pathophysiology, management strategies, and prognostic implications are encompassed within a single diagnostic category, limiting the predictive value of current definitions for long-term outcomes and therapeutic responsiveness (35) (Figure 1).

In this context, ongoing efforts have prioritized the identification of clinically meaningful phenotypes through comprehensive, multimodal assessment. Wu et al. described three principal phenotypes using a combination of imaging, echocardiographic, and bronchoscopy evaluations in infants with moderate-to-severe BPD (36). Building on these findings, Pierro et al. introduced additional phenotypic classifications, further highlighting the diverse disease processes encompassed by BPD (37). Together, these approaches underscore the concept of BPD as a syndrome comprising multiple, often overlapping phenotypes, each linked to predominant involvement of specific compartments within the developing lung.

Recognizing BPD as a heterogeneous, multidimensional syndrome has encouraged greater focus on phenotype-oriented management. Despite growing interest, strong evidence for truly individualized therapeutic strategies remains limited, and many interventions are still applied empirically. A key challenge in clinical practice is that BPD phenotypes rarely present in isolation; most infants demonstrate overlapping patterns or “mixed” phenotypes (38). These complex presentations are often associated with increased disease severity, prolonged respiratory support, and higher risks of morbidity and mortality. As a result, uniform treatment approaches have clear limitations. By adopting a phenotype-based framework, clinicians can better align therapeutic interventions with underlying mechanisms, offering the potential to improve symptom control and positively influence disease trajectory by targeting specific pathways of lung injury and repair.

## Parenchymal phenotype

The parenchymal phenotype is characterized by impaired distal lung development, with arrested alveolarization resulting in fewer, larger, and structurally simplified alveoli. Histologically, this is associated with diminished secondary septation and varying degrees of interstitial thickening, indicative of disrupted maturation of the alveolar–capillary interface. Functionally, these abnormalities manifest as reduced lung compliance, impaired oxygenation, and, in more severe cases, hypercapnia due to ventilation–perfusion (V/Q) mismatch and increased physiological dead space (39, 40).

This phenotype is considered a hallmark of contemporary BPD and reflects disruption of lung growth rather than purely postnatal injury. The resulting structural changes share similarities with emphysematous patterns observed in chronic obstructive pulmonary disease, although they arise from distinct developmental mechanisms (41). Imaging plays a central role in characterizing parenchymal involvement, with computed tomography–based scoring systems enabling quantification of hyperexpansion, emphysema-like changes, and interstitial abnormalities (42).

Management of the parenchymal phenotype include lung-protective ventilation strategies to reduce ventilator-induced lung injury (VILI), careful titration of oxygen exposure, judicious use of corticosteroids to attenuate inflammation, and optimization of nutrition and somatic growth to support lung development (4). At present, no therapies can reverse the structural abnormalities associated with alveolar simplification and emphysema-like changes. Management therefore focuses on creating conditions that facilitate endogenous repair and continued lung growth. Importantly, alveolarization extends beyond the neonatal period, continuing from approximately 36 weeks' gestation into early childhood through progressive alveolar multiplication. This developmental plasticity provides a basis for functional improvement over time, and many infants demonstrate gradual recovery with eventual weaning from respiratory support, although outcomes remain highly variable and are influenced by disease severity and coexisting phenotypes (43, 44).

## Interstitial phenotype

The interstitial phenotype reflects abnormalities within the mesenchymal compartment, with altered cellular differentiation leading to septal thickening and expansion of the interstitial space. These changes increase diffusion distance and impair lung mechanics, often resulting in a restrictive functional pattern. Interstitial injury may be further exacerbated by capillary leak associated with inflammatory conditions such as respiratory distress syndrome, infection, or VILI (37). Clinically, this phenotype is associated with reduced compliance, increased work of breathing, and prolonged dependence on supplemental oxygen, while imaging may demonstrate diffuse or patchy interstitial opacities (45).

Therapeutic strategies for the interstitial phenotype are largely extrapolated from general principles of lung-protective care and anti-inflammatory therapy, reflecting the absence of phenotype-specific trials. Management emphasizes gentle ventilation, early transition to noninvasive respiratory support, and meticulous oxygen titration to limit ongoing epithelial and endothelial injury. Postnatal corticosteroids have long been used to reduce pulmonary inflammation and facilitate extubation. Dexamethasone is effective in improving short-term respiratory outcomes, but concerns regarding adverse neurodevelopmental effects have led to more selective use, typically in infants who are difficult to wean from mechanical ventilation, with efforts to minimize cumulative exposure (46). Inhaled corticosteroids have been proposed as an alternative to reduce systemic effects, although evidence regarding their efficacy and safety remains inconsistent (40).

Interstitial injury is often amplified by increased capillary permeability associated with inflammatory states such as infection, respiratory distress syndrome, or VILI, and is functionally associated with restrictive physiology. Observations from adult interstitial lung disease suggest potential benefit from corticosteroid therapy in selected cases, leading to the hypothesis that infants with a predominant interstitial phenotype may derive greater benefit from anti-inflammatory strategies if identified early (37, 47). However, given the pleiotropic effects of glucocorticoids across multiple lung compartments, it remains uncertain whether therapeutic responses differ meaningfully between phenotypes.

## Congestive phenotype

The congestive phenotype is characterized by pulmonary fluid overload and interstitial edema, which contribute to impaired lung compliance and gas exchange. Preterm infants are particularly susceptible due to immature pulmonary lymphatic function and limited capacity for fluid clearance. The accumulation of interstitial fluid exacerbates V/Q mismatch and increases respiratory support requirements (37). Clinically, this phenotype is often dynamic, with fluctuations in oxygen and ventilatory needs in response to fluid status. Although chest radiography remains the most used modality for detecting pulmonary edema, its specificity is limited, and findings may overlap with other parenchymal abnormalities. Nonetheless, recognition of a congestive component is clinically relevant, as it may inform fluid management and the use of diuretic therapy (45).

Management of the congestive phenotype focuses on reducing pulmonary fluid overload and its impact on lung mechanics. Diuretics are commonly employed in the neonatal intensive care setting to decrease interstitial and alveolar edema, with short-term improvements in lung compliance and oxygenation frequently observed (4).

Despite these physiological benefits, evidence supporting sustained clinical improvement is limited, and the impact on long-term outcomes remains uncertain. Furthermore, prolonged use is associated with potential adverse effects, including electrolyte disturbances, nephrocalcinosis, and impaired growth, warranting a cautious and individualized approach. In selected infants with clear evidence of pulmonary edema, targeted diuretic therapy may alleviate symptoms and potentially influence aspects of disease progression (37).

## Vascular phenotype

The vascular phenotype represents a critical component of BPD pathophysiology and is a major determinant of adverse outcomes with recurrent hypoxemia, prolonged hospitalization, and ongoing respiratory support needs. The vascular abnormalities are closely linked to impaired alveolar development, reflecting the interdependence of vascular and parenchymal growth within the developing lung. Consequently, BPD is increasingly conceptualized as a disorder of the alveolar–capillary unit. Clinically, PH may emerge early in the postnatal course or develop later in infants with established BPD (48, 49).

Diagnosis is typically based on echocardiographic evidence of elevated right ventricular pressures or dysfunction, with biomarkers such as brain natriuretic peptide providing supportive information (50). Affected infants often exhibit prolonged oxygen dependency, recurrent hypoxemic episodes, and increased ventilatory requirements (45). Importantly, PH represents the most clinically significant manifestation of PVD and is strongly associated with increased morbidity and mortality, while abnormalities in gas exchange may further exacerbate vascular dysfunction (39).

Management of PVD, particularly when complicated by PH, is centered on reducing pulmonary vascular resistance and supporting right ventricular function. Initial management prioritizes optimization of fluid status and appropriate ventilatory support to minimize intrapulmonary shunting and secondary vasoconstriction. Pharmacological therapy primarily targets the reactive component of PH through vasodilation. Sildenafil is widely used and has been associated with improvements in pulmonary hemodynamics and oxygenation, while endothelin receptor antagonists and prostacyclin analogues may be considered in selected cases, although evidence in neonates remains limited. Inhaled nitric oxide may provide transient benefit in acute settings but has not demonstrated consistent long-term efficacy (2, 51). Importantly, these therapies do not address the fixed structural abnormalities of the pulmonary vasculature, including reduced vascular growth and abnormal architecture, which are central to BPD pathophysiology.

## Airway phenotype

Airway involvement is another major contributor to respiratory morbidity in BPD, encompassing both central and peripheral airway abnormalities arising from disrupted development, inflammation, and iatrogenic injury.

## Central airway disease

Central airway disease is characterized by structural immaturity and weakness of the trachea and main bronchi, manifesting as tracheomalacia, bronchomalacia, or subglottic stenosis (52). These conditions result in excessive airway compliance and dynamic collapse, particularly during expiration, leading to airflow limitation and increased work of breathing. The tendency for airway collapse is influenced by the balance between airway wall rigidity and transmural pressure gradients and is exacerbated in preterm infants by increased compliance and structural remodeling, including smooth muscle hypertrophy, epithelial inflammation, and airway wall thickening (53, 54). Central airway pathology is particularly common in infants with more severe BPD, likely reflecting prolonged exposure to intubation and mechanical ventilation. Diagnostic confirmation relies on bronchoscopy, while functional assessment may reveal characteristic expiratory flow limitation (55).

In central airway disease, such as tracheomalacia or bronchomalacia, ventilatory strategies aim to maintain airway patency and prevent dynamic collapse, often using higher levels of positive end-expiratory pressure (PEEP) or flow-based support. Severe cases may require prolonged respiratory support, tracheostomy, and home ventilation (36). Pharmacological approaches, including cholinergic agents, have been used, although evidence supporting their efficacy is limited (56). Structural complications related to prolonged intubation, such as subglottic stenosis, may necessitate surgical intervention. The natural history is often characterized by gradual improvement, with many cases resolving within the first two years of life, although long-term outcomes remain incompletely defined (39).

## Peripheral airway disease

Peripheral airway disease involves structural remodeling of the small airways, including inflammation, smooth muscle hypertrophy, and extracellular matrix deposition, leading to airway narrowing, increased resistance, and gas trapping. Clinically, this phenotype may resemble asthma, with wheezing and airflow obstruction; however, the inflammatory profile is often non-eosinophilic, and airflow limitation frequently includes a fixed component, resulting in variable responsiveness to bronchodilator therapy (40). Imaging may demonstrate hyperinflation and air trapping, and the degree of reversibility is heterogeneous. Notably, peripheral airway disease may also be present in preterm infants who do not meet conventional diagnostic criteria for BPD, suggesting that it represents a broader continuum of prematurity-associated lung injury (45).

Management of peripheral airway disease focuses on alleviating airflow obstruction and addressing airway reactivity. Bronchodilators and anticholinergic agents are frequently trialed, although responses are heterogeneous and often less pronounced than in classical asthma, reflecting the contribution of fixed structural changes (57, 58). The variability in response underscores the importance of individualized assessment, ideally incorporating objective measures of lung function when feasible, to guide therapy and minimize unnecessary treatment. Current practice varies widely across institutions, highlighting the need for more robust evidence to inform standardized approaches (59).

## Hemodynamic phenotyping of BPD and therapeutic implications

The main value of early hemodynamic assessment is not only to detect PH or PVD, but to define the predominant pattern of cardiopulmonary impairment. Using clinical data, echocardiography and, when available, invasive measurements, BPD-PH can be broadly grouped into three pathophysiological phenotypes (2, 7, 15). Type 1, pre-capillary phenotype, is dominated by an intrinsic increase in PVR due to remodeling of the immature pulmonary vasculature, including delayed vascular growth, EndoMT, smooth muscle proliferation and endothelial dysfunction. Left-to-right shunts are absent or small, and LV filling pressures are normal or only slightly elevated. Management therefore focuses on limiting ongoing lung injury, using pulmonary vasodilators judiciously, and supporting RV function, with the aim of reducing PVR and halting adverse vascular remodeling.

Conversely, type 2, flow-dependent phenotype, is driven primarily by pulmonary overcirculation from moderate-to-large left-to-right shunts. The main hemodynamic insult is increased volume and hydrostatic pressure in the pulmonary capillary bed, causing recurrent interstitial edema and perpetuating lung injury. PVR may initially be normal or only mildly raised, but repetitive mechanical stress can lead to secondary vascular remodeling. In this context, indiscriminate use of pulmonary vasodilators risks worsening overcirculation and edema. Treatment instead centers on careful management of PDA and other shunts, relative fluid restriction, and optimization of ventricular function.

Finally, type 3 BPD-PH, post-capillary phenotype, is characterized by pulmonary venous congestion and post-capillary PH due to LV systolic/diastolic dysfunction or pulmonary vein stenosis. Typical findings include elevated PCWP, increased E/e' and markers of left atrial hypertension. Clinically, these infants often have recurrent pulmonary edema that responds poorly to therapies aimed only at arterial pulmonary vasodilation. Here, the therapeutic target is reduction of LV filling pressures and correction of the post-capillary lesion: improving diastolic function, controlling systemic hypertension, considering ACE inhibitors when systemic afterload is a key driver, and instituting specific management for pulmonary vein stenosis. In this phenotype, isolated use of pulmonary vasodilators is usually ineffective and may aggravate congestion.

In summary, precise hemodynamic phenotyping helps avoid potentially harmful interventions, such as vasodilators in predominantly venous or flow-dependent PH, which can worsen pulmonary edema, and allows PDA management to be tailored to the functional role of the ductus at a given time, rather than following uniform treatment algorithms.

## Prognosis and outcomes

Infants with severe BPD, particularly those complicated by PH, continue to face a high burden of adverse outcomes, with mortality rates approaching 40%–50% within the first two years of life. Morbidity is similarly significant, as nearly half of affected infants require rehospitalization during the first year, most commonly due to respiratory infections and exacerbations of underlying lung disease (2, 60). Beyond the neonatal period, BPD is increasingly recognized as a chronic, multisystem condition with consequences that extend well into childhood and adulthood. Survivors frequently demonstrate asthma-like symptoms, exercise intolerance, emphysematous changes, and pulmonary vascular disease. Additional features include impaired gas transfer related to alveolar abnormalities, tracheobronchomalacia, reduced exercise capacity, and diminished respiratory reserve (61).

In parallel, BPD is strongly associated with adverse neurodevelopmental outcomes, including cognitive impairment, language delay, and cerebral palsy. These associations likely arise from a combination of shared antecedents related to extreme prematurity and the systemic effects of chronic lung disease, inflammation, and sustained hypoxemia (62). Individuals with a history of BPD, particularly those with antecedent PH, are at elevated risk for long-term cardiovascular complications, including right ventricular dysfunction and persistent pulmonary vascular disease (63).

The substantial heterogeneity that characterizes BPD highlights the urgent need for more precise diagnostic and therapeutic approaches. Emerging strategies—including advanced imaging modalities, comprehensive physiological assessment, and biomarker-driven techniques—offer promising avenues for improved phenotypic characterization and prediction of treatment response. Concurrently, the growing population of BPD survivors underscores the importance of structured, longitudinal follow-up and well-coordinated transition from pediatric to adult care, given the complex and evolving respiratory and cardiovascular needs that extend across the lifespan.

## Conclusion

Bronchopulmonary dysplasia is increasingly recognized as a chronic, heterogeneous cardiopulmonary syndrome rather than a purely respiratory diagnosis. Viewing BPD through a hemodynamic lens highlights how disturbed transitional circulation, impaired angiogenesis, pulmonary vascular remodeling and biventricular dysfunction converge on a shared pathway of elevated PVR, pulmonary edema and gas-exchange impairment. In this framework, BPD-associated pulmonary hypertension emerges not as an epiphenomenon but as a central determinant of prognosis, tightly linked to mortality, prolonged respiratory support and long-term cardiopulmonary sequelae.

Early hemodynamic screening using NT-proBNP and targeted neonatal echocardiography provides a window into this evolving disease process, allowing subclinical pulmonary vascular disease, RV/LV dysfunction and clinically relevant shunts to be identified within the first days of life. These tools enable risk stratification that goes beyond oxygen requirement, support phenotype-oriented classification, and open the door to precision management, whether by tailoring ventilatory strategies, optimizing fluid balance, reconsidering PDA treatment, or directing pulmonary vasodilator and inotropic therapy according to the predominant pre-, post- or flow-dependent phenotype.

Despite these advances, evidence for truly individualized, hemodynamically guided treatment remains limited, and most interventions are still applied empirically. Priority areas for future research include prospective validation of early hemodynamic markers as therapeutic targets, randomized trials of phenotype-based strategies, and long-term follow-up of survivors to define the impact of early cardiovascular interventions on adult pulmonary vascular and right heart outcomes. As the population of extremely preterm survivors continues to grow, integrating detailed hemodynamic assessment into both neonatal and longitudinal care pathways will be essential to reduce the burden of BPD-PH and to improve functional, cardiovascular and neurodevelopmental outcomes across the lifespan.
